# Less Is Better: How Nutrition and Low-Carbon Labels Jointly Backfire on the Evaluation of Food Products

**DOI:** 10.3390/nu13041088

**Published:** 2021-03-26

**Authors:** Yuanhao Huang, Xiaoke Yang, Xianguo Li, Qian Chen

**Affiliations:** 1 School of Business, Renmin University of China, Beijing 100089, China; 2019000720@ruc.edu.cn (Y.H.); lixianguo@rmbs.ruc.edu.cn (X.L.); 2 College of Management and Economics, Fujian Agriculture and Forestry University, Fuzhou 350002, China

**Keywords:** sustainable, low-carbon label, nutrition label, resource allocation, zero-sum bias, anticipated enjoyment

## Abstract

(1) Background: Labeling is one of the significant strategies to guide sustainable consumption behaviors. Nowadays, multi labels being displayed on the front-of-pack of food products is a common phenomenon. However, labels seldom operate solo, and competition or complement effects may be exerted on different labels. Therefore, the research objective is to explore the interaction effect when nutrition and low-carbon labels appear simultaneously; (2) Methods: Across four scenario-based experiments, including ice cream, yogurt, steak, and toast, this study manipulated the separate and joint occurrences of low-carbon and nutrition labels, the interaction effect of joint labels was tested, and the serial mediation model, which includes resource allocation and anticipated enjoyment of food consumption, was verified; (3) Results: Results show that people have a positive preference for the nutrition label and the carbon label, respectively, while these two labels working simultaneously attenuate the positive effect of the single label. When facing nutrition and carbon labels simultaneously, people would infer partial resources are allocated to healthy and environmental aspects so they have a lower anticipated enjoyment from food consumption. Thus, these two labels working simultaneously attenuate the positive effect of the single label, and consumers have a lower evaluation of food products. In addition, the joint backfire on the effect is only exerted on people with a higher level of zero-sum bias and only when joint labels have a high consistency of labels; (4) Conclusions: This study solved the contradictory problem of the joint effect of positive labels. The findings in this research contribute to promote sustainable food consumption. We suggest that similar labels should be avoided in the same front-of-pack of food, and manufacturers need to use ads to bring down consumers’ zero-sum bias.

## 1. Introduction

The function of food is much beyond to solve starvation, and researchers have taken interests on many other aspects of food recently, as a pillar of cultural identity [1], food safety and environmental pollution [2], nutrition intake [3], and greenhouse gas (GHG) emissions from food [4]. Especially, it is often ignored that food consumption is a main factor of environmental degradation [5]. For example, GHG emissions from agricultural production account for 30% of the whole anthropogenic emissions [6,7]. On the other hand, food consumption is highly related to individual and public health. Many studies found that current dietary patterns cause serious health problems such as cardiovascular diseases and diabetes [8,9]. Therefore, changing towards sustainable consumption patterns has received much attention in the new millennium [10,11,12], and further, sustainable diets strategies should take environment impact and health into consideration [13].

Food is the typical credence product, which means consumers are unable to verify some attributes of food, such as nutrients, safety, and other altruistic features [14]. Therefore, external cues are essential for consumers to make purchase decisions [15]. In the real market, nutrition labels containing information about nutrients (calories, protein, energy, fat, sugar, and salt) are the most common cue to guide purchase decisions. Another emerging label is the carbon label, which is often used by firms to highlight the environmental impact of the product, i.e., some beverage products in Japan illustrate the carbon emission of 100 mL through the carbon footprint label. Similarly, carbon footprint labeling is also shown in some beef and dairy products in European countries [16]. Previous studies have shown that nutrition and carbon labels individually are beneficial for sustainable food consumption switching [17,18,19,20]. However, for the simultaneous appearance of multi labels on the front-of-pack of food, effects of these labels need to be re-estimated, because competition is exerted in complex information. The competition between multiple labels may have influence on the overall evaluation of food by consumers [21].

Few studies have examined the joint effect of nutrition and carbon labels on consumers’ preference. Defrancesco et al. [22] examined the consumers’ preference for health and environmentally friendly attributes of Italian pasta, and the results showed only consumers with certain taste preference pay premium for the health attribute and were unwilling to pay for the environmental attributes. In a study focused on healthy and environmentally sustainable food choices, health and environmental attributes were adopted as levels under the logo and label attributes, and conclusions drawn were based on product and consumer division [23]. Another study focused on yogurt consumption showed that healthy and environmentally friendly attributes on label displays were valued by consumers [24]. However, prior studies fail to consider a reality that labels of products seldom operate in solo and determining the interaction effect between multiple labels of food on preference of consumers is of significance [25,26,27]. The central question posed in the current study is how the nutrition label and the carbon label jointly influence the preference of consumers. Therefore, the main objectives of this study are to analyze the different effects of the nutrition label and the low-carbon label on consumers’ food preferences when they appear separately and in combination. Meanwhile, this study further explores the potential psychological mechanism of consumers and the applicable boundary of this effect. Based on the above research objectives, we put forward the core hypothesis, that is, nutrition and carbon labels backfire on the consumers’ intention when purchasing food.

The remainder of the current study is organized as follows. The next section reviews the literature related to food labeling, zero-sum beliefs, anticipated enjoyment motivation, and the four hypotheses of this study are put forward. Then we carry out our four experiments in the section of research methods and materials to verify the four hypotheses proposed in this study. Finally, we discuss the results along with theoretical contributions, managerial implications, and limitations of this study.

## 2. Hypothesis Development

To achieve sustainable food consumption, sustainable information at the product level is indeed significant to consumers [28]. Beyond the health halo from nutritional labels, nowadays, the rising popularity of ethical cues of food have been emphasized by firms to satisfy the growing demand for a moral feature of food products [29], and environmental impact has been one of the crucial concerning aspects [28]. Therefore, consumers can be of significance in contributing to a sustainable society through choosing food products with environmentally friendly and healthy attributes [25]. Despite some studies that have focused on the environmentally friendly and nutritional attributes simultaneously [22,23,24], few studies have determined the joint effect of these two attributes. Understanding how consumers responding to the nutrition label and the carbon label is significant to promote sustainable consumption behaviors. Nutrition and carbon labels are both positive cues, thus a following question is whether consumers always infer positive cue as higher quality or preference.

Research on the positive effect of food labels has extended to a large scale [30,31,32,33]; however, most of the studies only presented the isolated effects of labels. Consumers exposed to multi labels simultaneously can hardly evaluate these highlighted features in isolation. Instead, multi features work together on people’s assessment of food [21]. However, limited literature has examined the joint effect of multi attributes of food. The research on the effects of multi attributes of product showed controversial conclusions. One common viewpoint is that multi attributes can enhance incremental utility [34,35]. In the study by Bagchi and Davis [36], the results showed that numerosity heuristics predict the effect exerted on consumers, and people infer a larger effect from higher numerosity. By extension, consumers regard a product with multi attributes attached to be higher value than that with a single attribute, and this bias is derived from numerosity heuristics. Firms are inclined to attach add-on features to enhance the perceived utility of the product itself, and they consequently charge higher markups [37,38]. However, a second viewpoint is pointed out that rich information can lead to negative purchase decisions when the information goes over the threshold [15]. For example, consumers may infer a company’s intended labeling schemes as implications of quality, even though the actual meaning of label is not related to quality [39]. Another research similarly concluded that the label may also lead to misestimating the quality of products [40].

The idea of this study is closer to the second idea about the effect of multi labels simultaneously, which is that rich information can lead to negative purchase decisions when the information goes over the threshold. First of all, this study assumes that when low-carbon labels and nutrition labels appear at the same time, they represent that rich information. However, the two rich and positive information labels may have some antagonistic relationship with taste quality. On the one hand, according to [39], intended green information may lead to a decrease in purchase intentions due to assuming lower quality. On the other hand, a stereotype of the healthy attribute is that healthy equals bland or not tasty, which induces a negative effect on consumers’ preference and actual taste experience [41]. Secondly, except for the isolated effect of attributes of food products, several studies also emphasized the importance of the joint effect of different attributes [27,42]. When considering the joint effect of two positive cues, the results show it is not always complementary to each other, and sometimes there may be a substitute effect between two positive quality cues [26,43,44]. Therefore, we think that when nutrition and low-carbon labels appear at the same time, they may lead to information overload in terms of positive attributes of food to consumers, thus negatively affecting consumers’ inferences about food quality, especially on the taste of foods. Finally, at present, there are more and more foods in the market that pay attention to both health and taste or pay attention to both being low carbon and taste. Therefore, consumers have a high threshold for the decline in taste quality caused solely by health or low-carbon information. Therefore, it is likely that when a food is labeled only nutritious or low carbon, consumers will not experience a decrease in purchase intention. Hence, we hypothesize:

**Hypothesis** **1** **(H1).** *Consumers will have lower purchase intention when there are positive food labels jointly (vs. individually). In other words, nutrition and carbon labels jointly backfire on the consumers’ intention when purchasing food.*

In order to better explore the adverse effect caused by joint labeling, we firstly explored the psychological mechanism behind the adverse effect of consumers. Therefore, we have specifically introduced two concepts from psychology, one is the zero-sum belief, the other is the anticipated enjoyment motivation. The joint backfire is derived from a lay theory about zero-sum, which means consumers perceive resource allocating between attributes, and a superior attribute is compensated by other inferior attributes [45]. External cues would impact taste perception [46]. Considering this lay theory about “healthy = untasty” [41], Newman, Gorlin, and Dhar [39] determined the backfire on the intended green enhancement of products to be that limited resources were assumed to be allocated to the green attribute even if there is no resource transfer. This implies that multiple positive labels may trigger the assumption of resource allocation compared with the single one. In other words, when nutrition labels or low-carbon labels appear alone, it is difficult for consumers to generate zero-sum beliefs because they do not exceed the threshold of information perception. However, when the two labels appear at the same time, consumers naturally generate this zero-sum belief because they exceed the perceived threshold of positive attributes. Therefore, this study suggests that consumers would more intensively infer resource allocation from taste to positive attributes when the positive labels appear jointly (vs. individual).

The transfer of resources under zero-sum beliefs largely comes from the positive enhancement of green and healthy attributes and the anticipated enjoyment of food tastes. Anticipated enjoyment motivation is one of the significant factors of consumers purchasing food [47], and a better taste develops higher anticipated enjoyment from food [48]. Thus, this study deduces that a lower evaluation of taste may lead to a lower perceived enjoyment, and then cause a reduction of purchase intentions of food [49]. Hence, this research builds a serial mediation model and hypothesizes that:

**Hypothesis** **2** **(H2).** *Consumers will infer that more resources of food are allocated to positive attributes rather than to taste, and more resources on the positive attribute would be further inferred as the food having a lower level of anticipated enjoyment when the positive food labels are jointly present (vs. individually). Resource allocation and anticipated enjoyment serve as a serial mediation model in the joint backfire.*

People also have different tendencies to make zero-sum inferences about resources. People have a higher zero-sum bias when they have a stronger zero-sum inference tendency for the resources of a product. People assume resource allocation from zero-sum bias [50], which means people perceive a competition for resource distribution even though there are unlimited resources. A former study has confirmed that people employed zero-sum bias to evaluate products [51]. In other words, consumers perceived that resources of a product are limited and in zero-sum competition, a specific feature of the product is compensated by other features. The results of Mai et al. [45] showed that people thought ethical products are less strong, because consumers perceived that environmentally friendliness or other socially beneficial attributes are a tradeoff for other attributes [52,53,54].

However, in the study by Meegan [50], zero-sum bias is more likely to be employed by some people rather than all humans. In other words, because individuals have different levels of zero-sum bias [55], the lay theory about resource allocation is not always appropriate for all consumers. This study predicts that people with a high level of zero-sum bias are more likely to assume resources are allocated from taste to positive cues, and consumers who are low in zero-sum bias are in contrast. We further assumed that when consumers have a low zero-sum bias, they may be relatively insensitive to the transfer of resources between different attributes in a product. Therefore, when they see positive attributes such as nutrition and low carbon labels, they would not think that they are created by enterprises at the expense of food taste. Hence, this study hypothesizes that:

**Hypothesis** **3** **(H3).** *Consumers’ zero-sum bias will moderate the effect of positive cues on purchase intention. Consumers in a high zero-sum bias will have lower purchase intention when the food labels are jointly presented (vs. individually). Conversely, consumers with a low zero-sum bias have no significant difference of purchase intentions and no significant difference between joint food labels (vs. individual).*

Depending on cue consistency theory [56], the joint effect of multi attributes is related to whether the features are consistent or not [57]. When attributes are consistent (such as both positive cues), the effect of these cues is additive [21]. Health and environmentally friendliness are two dimensions of sustainability, and both are relative virtues of food providing delayed benefits (sustainable consumption patterns) alongside immediate costs (a reduction of taste) [58]. Meanwhile, healthy and low-carbon attributes of food are both inferred as a reduction of taste and enjoyment, and this complement may have a stronger negative effect on consumers’ purchase intentions of food. On the contrary, when the two positive attributes are mutually orthogonal, different aspects of attributes may not generate additional zero-sum bias of resources. This study assumes the boundary condition of the joint backfire on the effect is the consistency of labels. Hence, this study hypothesizes that:

**Hypothesis** **4** **(H4).** *Consistency level of joint positive labels will moderate the effect of purchase intention. When joint labels are consistent, consumers will have lower purchase intentions compared to an individual one. When joint labels are inconsistent, consumers’ purchase intentions are not significantly different compared to the individual label.*

## 3. Materials and Methods

**Figure 1:**
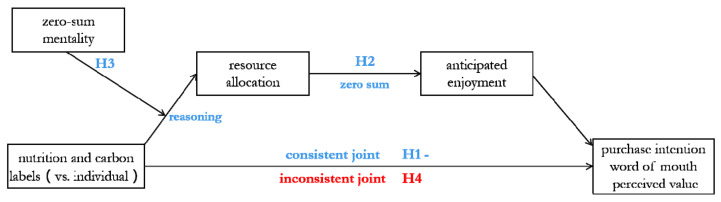
The main framework of the study.

In order to verify these four hypotheses, different stimulus materials were used in these four scenario-based experiments. By changing the test items of the experiment, potential factors and alternative explanations were eliminated, thus improving the external and internal validity of research conclusions. The main framework of the present study was shown in Figure 1.

In Study 1, the backfire on the effect (Hypothesis 1) was first verified by constructing an ice cream purchase scenario, and the intermediary mechanism (Hypothesis 2) was verified at the same time. Results proved that the resource allocation and anticipated enjoyment served as serial mediators in the joint backfire.

In Study 2, the conclusion stability of Hypotheses 1 and 2 was further enhanced. However, Study 2 is different from Study 1 in several aspects. On the one hand, the dependent variable increased the measurement of consumers’ word-of-mouth and perceived value. It proved the difference in consumers’ purchase intention for food from multiple dimensions. On the other hand, the benchmark group of no positive label was increased to compare the intergroup variations of resource allocation and anticipated enjoyment in joint, individual, and no positive label.

In Study 3, the boundary condition of the joint backfire, namely, consumers’ zero-sum bias (Hypothesis 3), was verified. At the same time, the healthy and low-carbon scenarios of positive labels were extended to the organic and country of origin (COO) scenarios through the consumption scenario of whole-cut steaks.

In Study 4, another serial boundary condition of the joint backfire, consistency level of joint positive labels (Hypothesis 4), was verified. At the same time, combined with the consumption scenario of toast bread, the potential intermediary explanation mechanism of conceptual fluency was eliminated.

### 3.1. Study 1: Joint vs. Individual Positive Labels

The backfire on the effect (Hypothesis 1) was verified in Study 1 and was explained from the perspectives of zero-sum and anticipated enjoyment motives (Hypothesis 2). Therefore, a serial mediation model (joint labels→resource allocation anticipated enjoyment purchase intention) was predicted. The intergroup design of joint carbon-nutrition labels, individual carbon label, and individual nutrition label for comparison were used in this experiment. Our expectation was that the joint group has the joint backfire compared to the two individual groups. However, there is no difference between the two individual groups, which eliminates the potential for carbon and nutrition labels to produce different consumer perceptions. In addition, we would also verify that consumers do not have different emotional states when they face the single label. Finally, in order to test whether the subjects really read the experimental stimulus and understood the content of the label, we asked the subjects to recall the label content of the stimulus and fill in the blanks at the end of the experiment, so as to exclude the careless subjects.

#### 3.1.1. Method

The Credamo online platform as a professional data survey platform has provided services to top universities around the world. Besides, Credamo has been used as a source of data acquisition for a number of authoritative journal articles [59]. In this research, 140 high-reliability respondents (Users with a credit score above 90 on the Credamo online) were randomly recruited from the Credamo online and were randomly assigned to one of the following three experimental groups: Joint carbon–nutrition group, individual carbon group, and individual nutrition group. Through random sample processing, the characteristics of the subjects in different experimental groups were kept as consistent as possible to reduce the impact of the demographic characteristics of the subjects on the experimental results. After checking whether there is a “Z” law i.e., most of the items are the extreme value 1 or 7, or those who failed to recall the labels, 4 samples were excluded and 136 valid questionnaires (the demographic characteristics of the specific samples are shown in Table 1) were recovered, including 46 respondents in the joint nutrition–carbon group, 43 respondents in the individual nutrition group, and 47 respondents in the individual carbon group. According to the paradigm of consumer behavior research, the effective sample size of each group above 40 meets the requirements of consumer behavior experiment [60].

#### 3.1.2. Procedure and Materials

**Figure 2:**
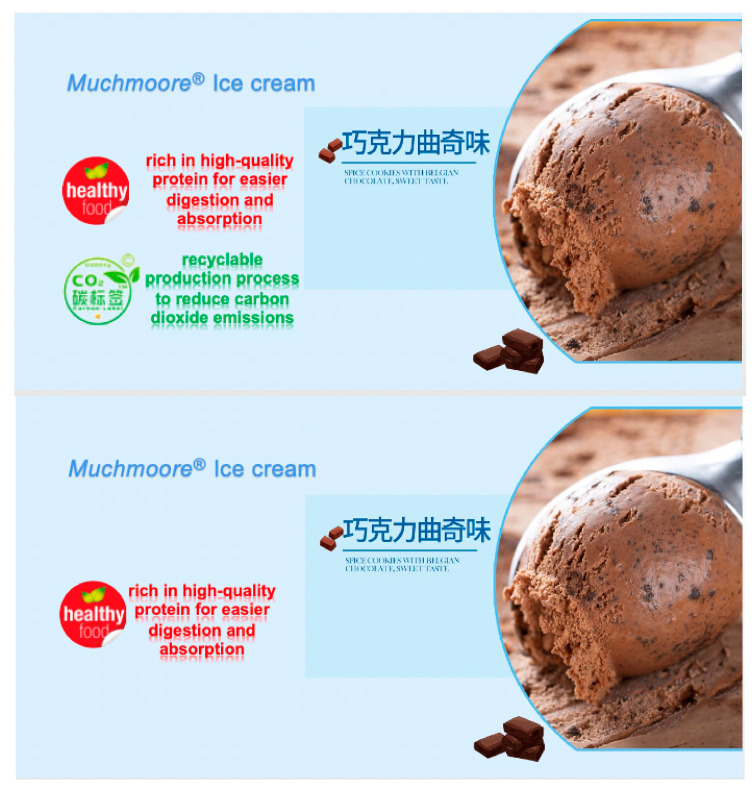

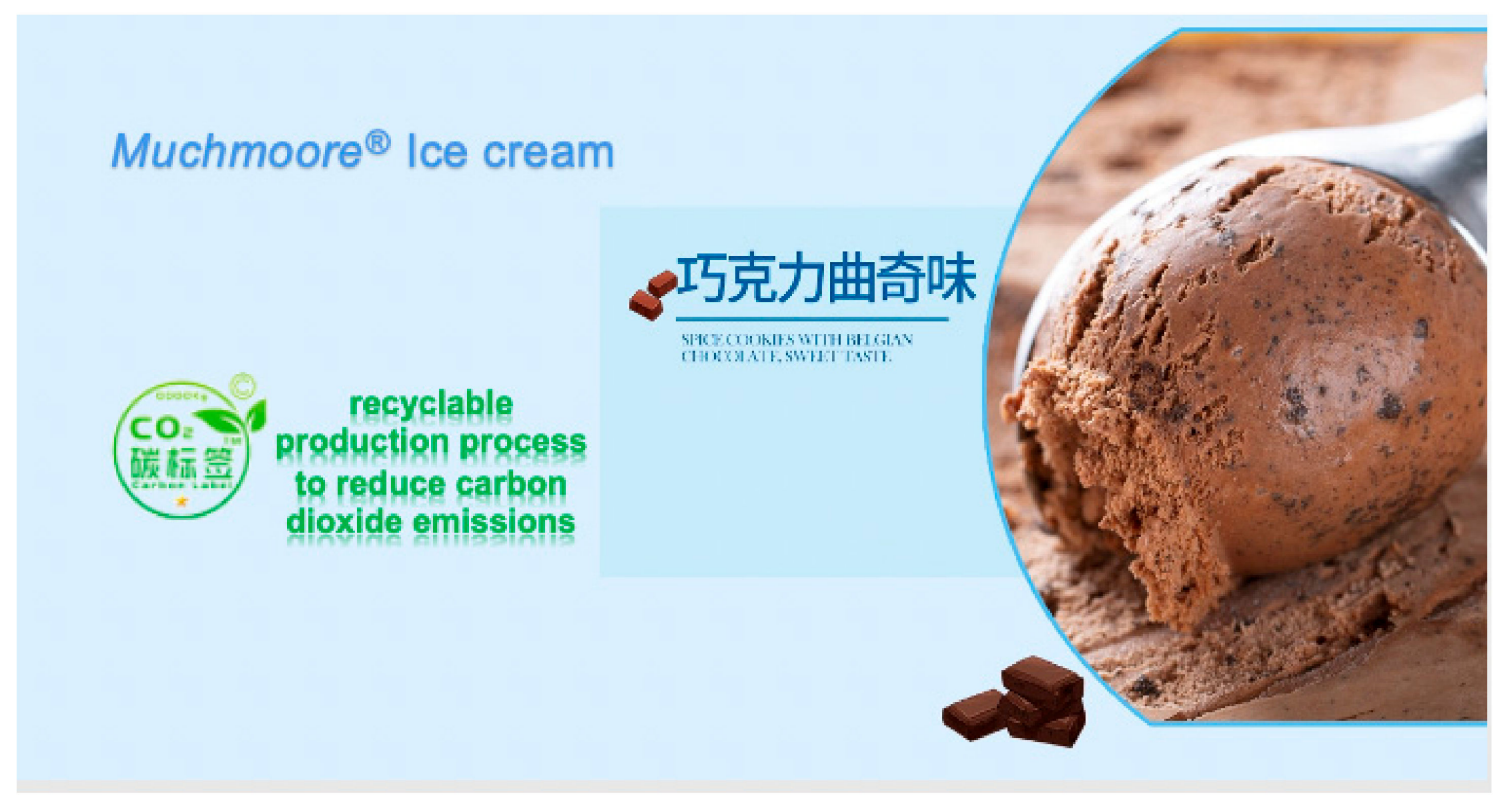
Labels in study 1.

Participants were randomly assigned to one of these three groups and should read the material of this ice cream, and accordingly evaluate and score. First of all, for the participants, they were presented with the background and stimuli of their own group. The background story of the experiment is that a food company is about to launch a new fictitious ice cream of “Muchmoore” (80 g/cup, 8 yuan). The carbon and nutrition labels and their brief introduction were attached to the product introduction of ice cream. The product introduction of the three groups of ice cream is different. The details are as follows: For individual nutrition cue, the product’s positive attribute was described as “rich in high-quality protein for easier digestion and absorption”. For the individual carbon cue, it was described as “recyclable production process to reduce carbon dioxide emissions”. The joint group added both nutrition and carbon labels, and two individual groups added nutrition or carbon label (Figure 2).

After reading about this ice cream, participants were asked to answer a series of questions to express their intentions and attitudes toward ice cream products. Participants’ purchase intentions were finally indicated by a 7-point scale (1 = definitely would not buy, 7 = definitely would buy). Participants firstly evaluated the resource allocation for product attributes through their agreement with the statement: “Do you think that in order to make the ice cream healthier, the company took resources away from making this product better tasting?” (1 = not at all, 7 = very much so). This measurement item was adapted from Newman (2014) [39] and modified moderately according to the study scenario. Participants also evaluated the anticipated enjoyment for this ice cream through the 3-items: “This ice cream is tasty?”, “Eating this ice cream will give me pleasure?”, and “I will enjoy eating this ice cream?” (1 = not at all, 7 = very much so). This measurement item was adapted from Gomez and Torelli (2015) [1] and modified moderately according to the study scenario. In addition, positive and negative effects of participants were also reported by a 7-point scale. Items of positive effect include “enthusiast”, “interested”, and “excited”, while items of negative effect include “upset”, “offended”, and “irritable”.

#### 3.1.3. Results

**Figure 3:**
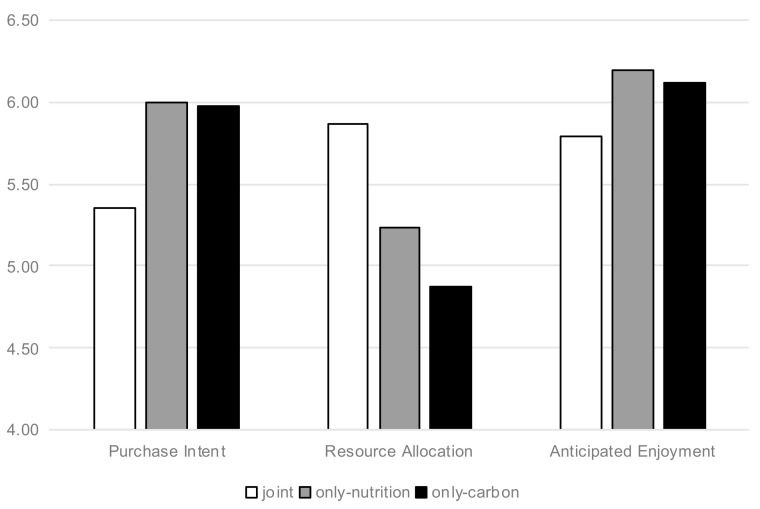
Backfire on the effect of labels on purchase intention, resource allocation, and anticipated enjoyment (Ice Cream).

Purchase Intention. The results of this study are depicted in Figure 3. A one-way Analysis of Variance (ANOVA) revealed the backfire on the effect of joint (vs. individual) positive labels on the purchase intention (F(2, 133) = 7.091, *p* = 0.001). F value represents the results of F test on the purchase intention of the three groups, which proves that the three groups do not belong to the same normal distribution in terms of purchase intention and have significant differences. We further analyzed the mean values of “joint” group and the other two groups by t-test. The results showed that: Consumers showed lower purchase intention when the joint (vs. individual) labels on the front-of-pack of ice cream (M_joint_ = 5.35, Standard deviation (SD) = 1.1 vs. M_only-carbon_ = 5.98, SD = 0.847, t(91) = 3.104, *p* = 0.003, and M_only-nutrition_ = 6, SD = 0.845, t(87) = 3.121, *p* = 0.002). However, consumers’ purchase intentions had no significant difference between individual nutrition and carbon groups (t(88) = 0.119, *p* > 0.1). As can be seen from the results, the average values of “joint” group were significantly lower than those of the other two groups, and the difference was proved to be significant by the t-test. The results provide preliminary support for Hypothesis 1.

Resource Allocation. The data analysis idea is the same as above. A one-way ANOVA revealed a significant effect of joint (vs. individual) positive labels on the resource allocation for the ice cream, F(2133) = 6.546, *p* = 0.002. Consumers perceived higher resource allocation when the labels of ice cream were joint (vs. individual) (M_joint_ = 5.87, SD = 0.98 vs. M_only-carbont_ = 4.87, SD = 1.498, t(91) = 3.79, *p* = 0.001 and M_only-nutrition_ = 5.23, SD = 1.493, t(87) = 2.394, *p* = 0.019). However, the resource allocation had no significant difference between individual nutrition and carbon groups (t(88) = 1.141, *p* > 0.1). 

Anticipated Enjoyment. Three items related to anticipated enjoyment of this ice cream were highly correlated (α = 0.727). The α value represents that these three items can reasonably measure the expected enjoyment, and the reliability of simultaneous measurement is high. A one-way ANOVA revealed a backfire on the joint (vs. individual) positive labels of the anticipated enjoyment from this ice cream, i.e., F(2133) = 4.059, and *p* = 0.019. In addition, it is further inferred that the respondents had a lower level of anticipated enjoyment when the food labels were joint (vs. individual) (M_joint_ = 5.79, SD = 0.851 vs. M_only-carbon_ = 6.121, SD = 0.675, t(91) = 2.133, *p* = 0.036 and M_only-nutrition_ = 6.194, SD = 0.652, t(87) = 2.572, *p* = 0.012). However, the anticipated enjoyment also had no significant difference between individual nutrition and carbon groups (t(88) = 0.523, *p* > 0.1).

**Figure 4:**
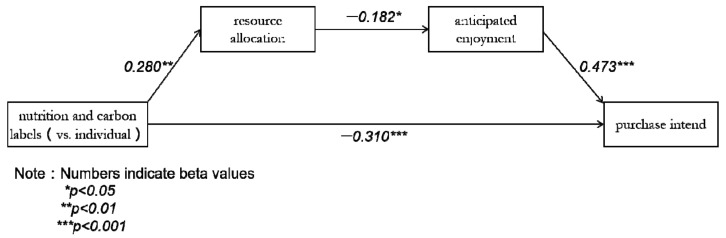
Study 1: The mediating effects of resource allocation and anticipated enjoyment in the relationship between positive labels and purchase intention.

Mediation Analysis. As can be seen from the above analysis of purchase intention, resource allocation, and anticipated enjoyment, there was no significant difference between the individual nutrition and carbon groups. Therefore, labels were coded as 1 = joint labels, and 0 = individual nutrition or carbon label. Measures of resource allocation and anticipated enjoyment were then taken as mediators of the effect of joint labels on the purchase intention. In addition, the specific predicted pathway was tested by a serial mediation model. The analysis of the mediating effect of the model was carried out strictly according to the behavioral paradigm [39]. To be specific, a bootstrap analysis with 5000 samples using the Model 6 (Model 6 means that it has one independent variable, one dependent variable, and two mediation variables with a continuous relationship) indicated that the full serial mediation model using both resource allocation and anticipated enjoyment was significant for this ice cream (indirect effect = 0.0622, SE = 0.0276, and 95% CI = −0.1377 to −0.023) [61]. The interval range of CI value does not contain 0, indicating that the mediating effect is significant [62]. However, additional analyses indicated that the “reverse” model (joint labels → anticipated enjoyment → resource allocation → purchase intention) had no significant effect for the ice cream (indirect effect = 0.0119, SE = 0.014, 95% CI = −0.0052 to 0.0555). The interval range of CI value includes 0, which means that the mediating effect is not significant when the two mediations are in reverse order. With further analysis, through the regression coefficient, we also verified the mediation effect again, and found that all the paths were significant (all *p* < 0.05) and the direction was consistent with the expectation (Figure 4). The results provide preliminary support for Hypothesis 2.

Control Variable. Three items related to positive and negative effects were highly correlated (α = 0.862 and 0.892, respectively). The positive effect had no significance on joint (vs. individual) positive labels (F(2133) = 1.847, *p* > 1), while the negative effect also had no significance on joint (vs. individual) positive labels (F(2133) = 1.607, *p* > 1).

### 3.2. Study 2: Benchmark Group Without Labeling

By replicating the process of Study 1, the core hypothesis (Hypothesis 1 and 2) was verified again. Unlike Study 1, the benchmark group of no label was added in Study 2 to compare with the relative variations of three groups. The intergroup design of joint carbon and nutrition labels, individual nutrition label, and no label was used for comparison. In addition, consumers’ behavioral factors were enhanced by adding the measurement of word-of-mouth and perceived value for food.

#### 3.2.1. Method

One hundred and sixty high-reliability respondents were recruited from the Credamo online platform and were randomly assigned to one of the following three experimental groups, i.e., joint carbon-nutrition group, individual nutrition group, and no positive label group. After checking whether there is a “Z” law or those who failed to recall the labels, 13 samples were excluded, and 147 valid questionnaires were recovered, including 48 respondents in the joint group, 45 respondents in the individual nutrition group, and 54 respondents in no positive labeling group.

#### 3.2.2. Procedure and Materials

**Figure 5:**
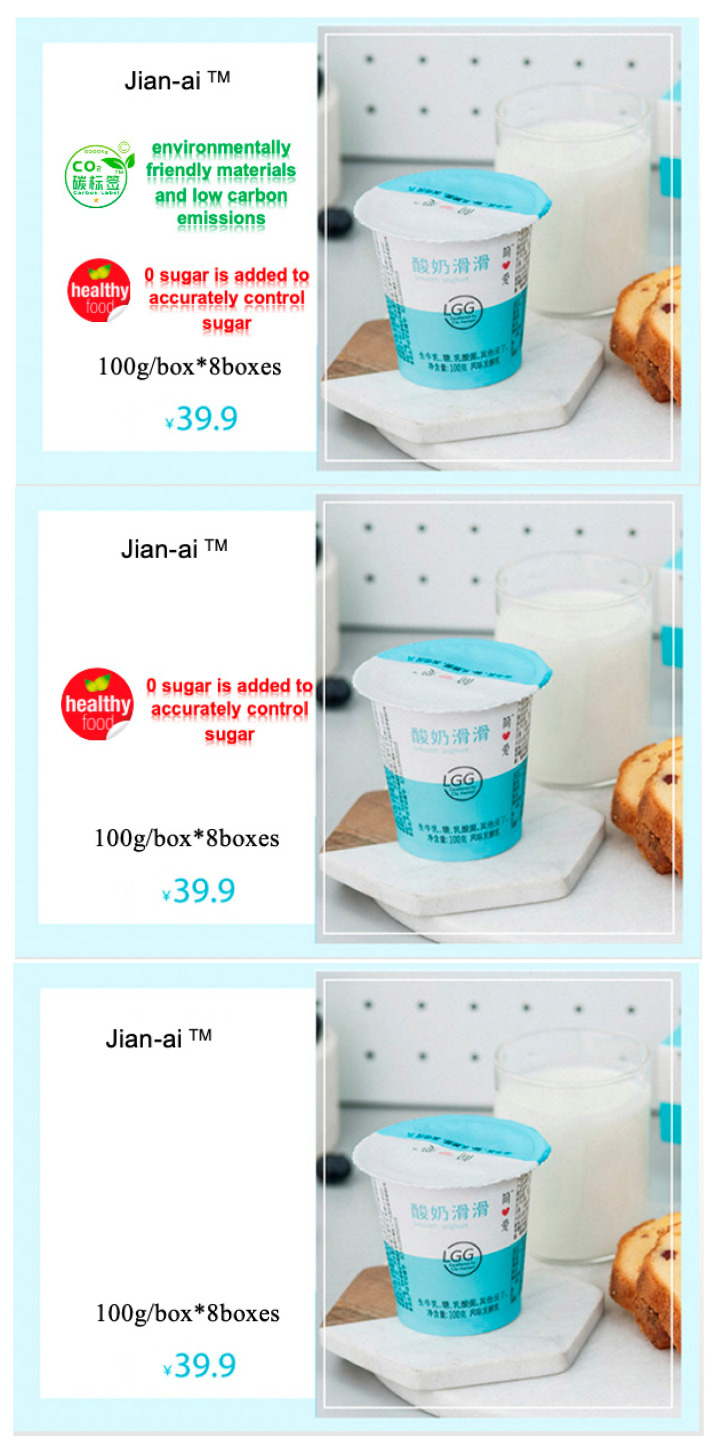
Labels in study 2.

The experimental procedure of study 2 was the same as that of study 1. Participants were asked to read the material of yogurt product, and accordingly evaluate and score. The background story of experiment was that a food company is about to launch a new fictitious yogurt product of “Jian-ai” (100 g/box ∗ 8 boxes, 39.9 yuan). The positive labels are mainly divided into nutrition and carbon aspects. For the nutrition label, the product’s positive attribute was described as “0 sugar is added to accurately control sugar”. For the carbon label, it was described as “environmentally friendly materials and low carbon emissions”. The joint group added both nutrition and carbon labels. The individual nutrition group only added the nutrition label, and no-label group had neither nutrition nor carbon label (Figure 5). Participants were randomly assigned to one of these three groups.

For this yogurt experiment, participants firstly evaluated the purchase intention for product attributes, and then evaluated the resource allocation and anticipated enjoyment for yogurt. Their word-of-mout, and perceived value were finally indicated by a 7-point scale. The item of word-of-mouth for this yogurt is “I recommend this yogurt to someone who wants to buy dairy products” (1 = not at all, 7 = very much so). This measurement items were adapted from Lee et al. (2010) [63]. Perceived value for this yogurt includes 4 items, i.e., “I think this yogurt is well made”, “I think the quality of this yogurt is acceptable”, “I think this yogurt is worth the money”, and “I think this kind of yogurt is economic” (1 = not at all, 7 = very much so). These measurement items were adapted from Sweeney and Soutar [64].

#### 3.2.3. Results

**Figure 6:**
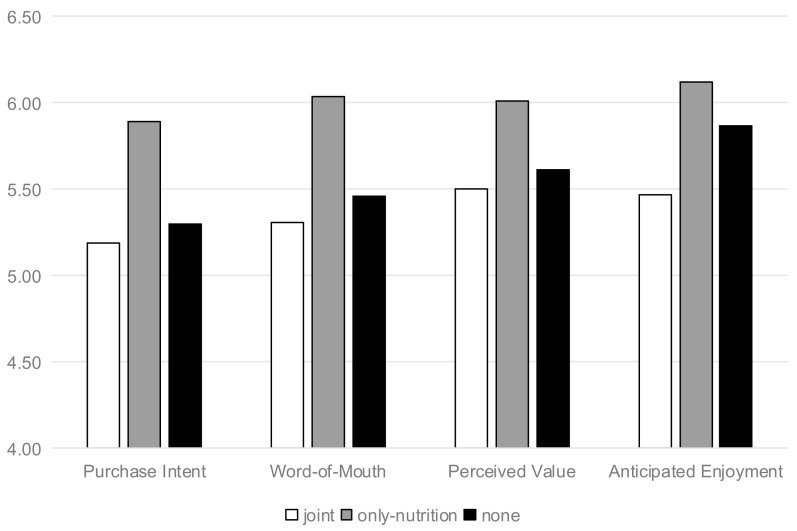
The backfire on the effect of cues on the purchase intention, word-of-mouth, perceived value, and mediators (Yogurt).

Purchase Intention. The data analysis method of the results of study 2 was the same as that of study 1. Results of this study were depicted in Figure 6. A one-way ANOVA revealed the backfire on the effect of joint labels (vs. individual) on the purchase intention for the yogurt (F(1133) = 7.091, and *p* = 0.001). Consumers had lower purchase intention when the yogurt labels were joint (vs. individual) (M_joint_ = 5.35 SD = 1.1 vs. M_only-nutrition_ = 5.98, SD = 0.847, t(91) = 3.104, *p* = 0.003). However, consumers’ purchase intention had no significant difference between “joint” group and “none” group (t(88) = 0.119, *p* > 0.1). In the same way, consumers had lower word-of-mouth (t(91) = 3.104, *p* = 0.003) and perceived value (t(91) = 3.104, *p* = 0.003) when the yogurt labels were joint (vs. individual). By analyzing the purchase intention, word-of-mouth, and perceived value, it could be seen that the values of these three variables were highest in the “individual” group. For these three variables, the joint group had no significant difference from the benchmark group, showing a potential inverted “U”. Among them, the “individual” group is the peak value of inverted “U”, while “joint” group and “none” group are the two ends of inverted “U”. It was indicated that the joint positive labels generated the joint backfire.

Resource Allocation. A one-way ANOVA revealed the significant effect of joint labels (vs. individual) on the resource allocation (F(1133) = 6.546, *p* = 0.002). Consumers perceived higher resource allocation when labels were joint (vs. nutrition cue and no positive cues) (M_joint_ = 5.87, SD = 0.98 vs. M_only-nutrition_ = 4.87, SD = 1.498, t(91) = 3.79, *p* = 0.001 and M_none_ = 5.23, SD = 1.493, t(87) = 2.394, *p* = 0.019). However, the resource allocation had no significant difference between “individual nutrition” group and “none” group (t(88) = 1.141, *p* > 0.1). In further analysis of the results, compared with the “joint” group, the resource allocation generated by “individual” group was lower, which had no significant effect on the subsequent anticipated enjoyment level. 

Anticipated Enjoyment. Three items related to anticipated enjoyment were highly correlated (α = 0.727). A one-way ANOVA revealed the backfire on the effect of joint labels (vs. individual) on the anticipated enjoyment (F(1133) = 4.059, *p* = 0.019). In addition, consumers perceived lower anticipated enjoyment when the food labels were joint (vs. nutrition and no positive cues) (M_joint_ = 5.87, SD = 0.98 vs. M_only-nutrition_ = 4.87, SD = 1.498, t(91) = 3.79, *p* = 0.001 and M_none_ = 5.23, SD = 1.493, t(87) = 2.394, *p* = 0.019). However, the anticipated enjoyment had no significant difference between the “individual nutrition” group and “none” group (t(88) = 0.523, *p* > 0.1).

Mediation Analysis. As in Study 1, we analyzed the mediation model of this study. The full serial mediation model using both resource allocation and anticipated enjoyment was significant (indirect effect = −0.1133, SE = 0.0487, 95% CI = −0.2172 to −0.0260). However, additional analyses indicated that the “reverse” model had no significance (CI = −0.0102 to 0.0101). Similarly, replacing the purchase intention with word-of-mouth (indirect effect = −0.1259, SE = 0.0523, 95% CI = −0.2325 to −0.0291) and perceived value (indirect effect = −0.1097, SE = 0.0440, 95% CI = −0.1988 to −0.0278), the full serial mediation model was significant.

### 3.3. Study 3: Boundary Conditions for Consumer Characteristics

Hypothesis 3 was verified to provide an important boundary condition for the main effect in Study 3, i.e., consumers’ zero-sum bias. At the same time, the zero-sum bias, as a moderating variable, also was used as a theoretical aid by adjusting the intermediary to verify the intermediary logic of resource allocation anticipated enjoyment rather than a reverse way. In addition, the floodlight method was used to verify the important boundary effect of consumers’ zero-sum bias. Moreover, the nutrition and carbon labels in Studies 1 and 2 were extended to the scenarios of organic and COO to further verify the external validity of the main effect. In other words, we hope that the backfire on the effect proposed in this paper can be generated not only under nutrition and low-carbon labels, but also under similar labels, such as origin and COO labels. Therefore, the 2 × 2 mixed design was used, including two intergroup favorite cues (joint: Organic + COO vs. individual: Organic) and two intragroup zero-sum biases (strong vs. weak). In addition, the potential effect of consumers’ background knowledge was excluded in the experiment.

#### 3.3.1. Method

In the experiment, 140 high-reliability respondents were recruited from the Credamo online platform and were randomly assigned to one of the following two experimental groups: Joint organic-COO group, and individual organic group. The zero-sum bias was classified according to the evaluation of subjects, containing high zero-sum bias whose scores were higher than the average, and low zero-sum bias whose scores were lower than the average. After checking whether there is a “Z” law among them or those who failed to recall the labels, 11 samples were excluded and 129 valid questionnaires were recovered, including 61 respondents in the joint organic-COO group and 68 respondents in the individual organic group.

#### 3.3.2. Procedure and Materials

**Figure 7:**
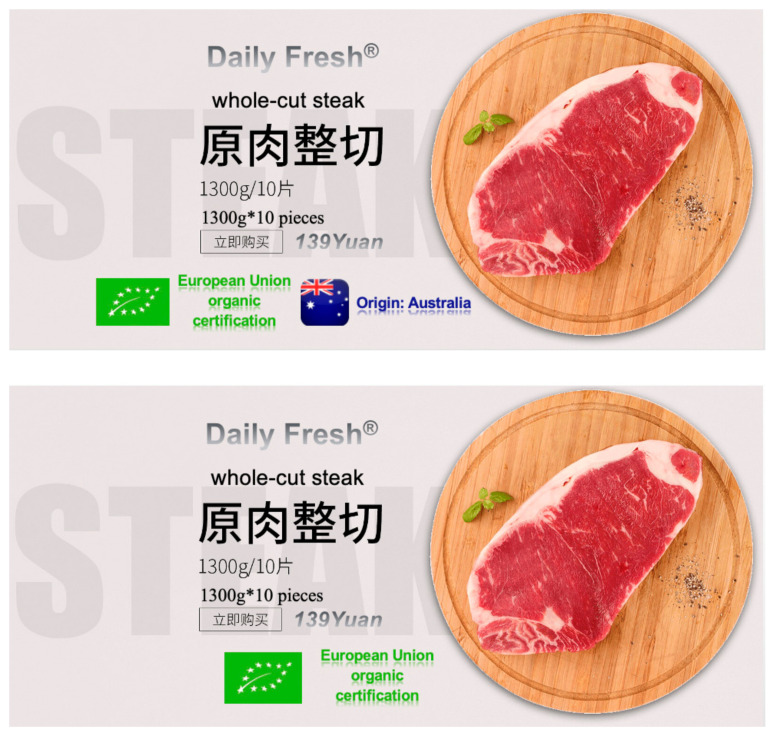
Labels in study 3.

Participants were asked to read the material of whole-cut steak, and accordingly evaluate and score. The background story of this experiment is that a food company is about to launch a new whole-cut steak of “Daily Fresh” (1300 g × 10 pieces, 139 yuan). In the organic label, the product’s positive attributes were described as “Certified organic by the European Union”. In the COO label, the label was described as “Origin: Australia”; the joint group added both organic and COO labels while the individual organic group only added the individual organic label (Figure 7). Participants were randomly assigned to one of these groups.

For the steak product, participants firstly evaluated purchase intention for product attributes. Participants’ resource allocation and anticipated enjoyment were also indicated, and finally the zero-sum bias was evaluated. The items of zero-sum bias include “I think people are more likely to excel at something when they put all their effort into it”, “I think it is better to gain professional knowledge by focusing on one skill rather than dabbling in many skills”, “I think if a product claims to have multiple functions, it is likely that each function is not very effective”, and “I think, if a product claims to provide only one function, then it is more likely to be the best in this function” (1 = not at all, 7 = very much so). These measurement items were adjusted from Różycka-Tran, Boski, and Wojciszke [55] and Burleigh et al. [65]. In addition, participations were asked to report their information of organic products and original countries. The measurement item “I know a lot about the organic food or the prime country for steak production” (1 = not at all, 7 = very much so) was referenced from Hoek, Pearson, James, Lawrence, and Friel [23].

#### 3.3.3. Results

**Figure 8:**
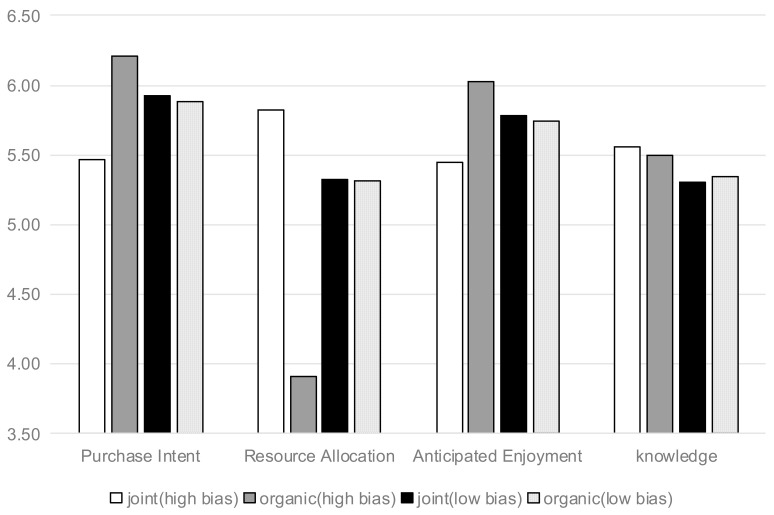
Backfire on the effect of joint positive cues in a situation of different zero-sum bias (Steak).

Purchase Intention. Results of Study 3 were depicted in Figure 8. Through a two-way ANOVA, a significant interaction was revealed between the organic and COO labels (joint vs. individual) and the zero-sum bias (high vs. low) (F(1125) = 4.751, *p* = 0.031). In the context of high consumers’ zero-sum bias, consumers had lower purchase intention when the food labels were joint (vs. individual) (M_joint_ = 5.47, SD = 1.134 vs. M_only-organic_ = 6.21, SD = 0.978, t(66) = 2.863, *p* = 0.006). However, in the context of low consumers’ zero-sum bias, consumers’ purchase intention had no significant difference (M_joint_ = 5.93, SD = 0.958 vs. M_only-organic_ = 5.88, SD = 0.946, t(59) = 0.178, *p* > 0.1).

Resource Allocation. Through a two-way ANOVA, a significant interaction was revealed between the organic and COO labels and the zero-sum bias (F(1125) = 14.039, *p* = 0.001). In the context of high consumers’ zero-sum bias, consumers perceived higher resource allocation when the food labels were joint (vs. individual) (M_joint_ = 5.82, SD = 0.936 vs. M_only-organic_ = 3.91, SD = 1.96, t(66) = 5.132, *p* = 0.001). However, in the context of low consumers’ zero-sum bias, consumers’ resource allocation had no significant difference (M_joint_ = 5.33, SD = 1.209 vs. M_only-organic_ = 5.32, SD = 1.387, t(59) = 0.178, *p* > 0.1).

Anticipated Enjoyment. This was similar to the former two analyses. A significant interaction was revealed between the two positive labels (organic and COO) and the zero-sum bias (F(1125) = 4.386, *p* = 0.038). In the context of high consumers’ zero-sum bias, consumers perceived lower anticipated enjoyment when the food labels were joint (vs. individual) (M_joint_ = 5.451, SD = 0.836 vs. M_only-organic_ = 6.029, SD = 0.78, t(66) = 2.949, *p* = 0.004). However, in the context of low consumers’ zero-sum bias, consumers’ anticipated enjoyment had no significant difference (M_joint_ = 5.778, SD = 0.686 vs. M_only-organic_ = 5.745, SD = 0.947, t(59) = 0.151, *p* > 0.1).

Mediation Analysis. A moderated mediation analysis was conducted to test the predicted relationship. To be more specific, positive labels were taken as the predictor variable, and the zero-sum bias and anticipated enjoyment were taken as the moderator and mediator. The bootstrap analysis with 5000 samples using the Model 7 (moderated mediation) indicated that the full model was significant (R^2^ = 0.1929, and *p* = 0.0001). In addition, the P value represents that the model is significant, while R2 represents that the model’s fit degree is good. In further analysis, the anticipated enjoyment mediated the effect of joint positive labels on purchase intention for high zero-sum bias (indirect effect = 0.2889, SE = 0.1522, 95% CI = 0.0607 to 0.6494), rather than low zero-sum bias (indirect effect = −0.0163, SE = 0.1071, 95% CI = −0.1994 to 0.2247). The results provide support for Hypothesis 3.

Since a combined serial and moderation analysis was not permitted in the model above, a serial mediation analysis on the resource allocation and anticipated enjoyment was further conducted. In the context of high consumers’ zero-sum bias, the full serial mediation model was significant (indirect effect = 0.1205, SE = 0.0861, 95% CI = 0.0026 to 0.3363), and the “reverse” model was not significant (indirect effect = 0.0085, SE = 0.0211, 95% CI = −0.0347 to 0.0528). Thus, the mediation order logic of resource allocation anticipated enjoyment was verified, which was consistent with a serial mediation analysis of Studies 1–3. However, in the context of low consumers’ zero-sum bias, this model was not significant (indirect effect = 0.0001, SE = 0.0121, 95% CI = −0.0205 to 0.0295).

Zero-sum bias. The moderating effect of consumers’ zero-sum bias was further examined. Because the moderator was a cardinal variable, the Johnson–Neyman floodlight analysis technique [66] was used to examine the backfire across the entire range of consumers’ zero-sum bias values. The PROCESS Model 1 [67] (Model 1 represents the test of causality between independent variables and dependent variables under different moderating variables) was used to run the floodlight analysis. PROCESS is a tool for path analysis-based moderation and Model 1 contains independent, moderator, and dependent variables. With 95% CI, the backfire on the effect of joint positive labels was significant for zero-sum bias values of more than 5.0791 (52.71% of the participants) and less than 1.6123, and the values between 5.0791 and 1.6123 had no significance. However, those with a value below 1.6123 only accounted for 0.7752% of the total population. Therefore, the effect was out of operation (*p* > 0.05) for a zero-sum bias value of below 5.0791 (47.29%). The overall interaction effect was significant (*p* = 0.0067), which supported Hypothesis 3. In a nutshell, in the context of low zero-sum bias of consumers (<5.0791), consumers’ purchase intention had no significant difference between joint food labels (vs. individual). When higher than 5.0791 (52.71%), it could be regarded as a high zero-sum bias consumer. The floodlight method provided a zero-sum bias threshold for subsequent related research.

Control Variable. The background knowledge of COO or organic label had no significant interaction between the labels and the zero-sum bias (F(1125) = 0.198, *p* > 0.1). Four conditions were coded, i.e., 11 = joint labels and high zero-sum bias, 00 = individual organic label and low zero-sum bias, 10 = joint labels and low zero-sum bias, and 01 = individual organic label and high zero-sum bias. The mean values between groups were as follows: M_11_ = 5.56, SD = 0.963, M_00_ = 5.35, SD = 1.07, M_10_ = 5.3, SD = 1.171, and M_01_ = 5.5, SD = 1.308. As indicated by the data, there was no significant difference in the mean of each group.

### 3.4. Study 4: Boundary Conditions for Labels’ Characteristics

In Study 4, the new scenario was used to verify the main effect again. Furthermore, from the perspective of product labels, an important boundary condition, the consistency of joint labels, was further provided for the main effect to verify the Hypothesis 4. This boundary condition could expand the application scope of joint positive labels in actual applications. Because the individual group has only one label, there was no distinction between high or low level of consistency. Three intergroup designs were used: Highly consistent nutrition and carbon labels, individual carbon label, and low-consistency service and carbon label. At the same time, the difference in the conceptual fluency of consumers caused by different types or combinations of labels were ruled out and the stability of internal mechanism of main effect was further tested.

#### 3.4.1. Method

One hundred and fifty high-reliability respondents were recruited from the Credamo online platform and randomly assigned to one of the following three experimental groups: Carbon–nutrition group, individual carbon group, and carbon–service group. After checking whether there was a “Z” law among them or those who failed to recall the labels, 14 samples were excluded and 136 valid questionnaires were recovered, including 44 respondents in nutrition–carbon group, 45 respondents in the individual carbon group, and 47 respondents in service–carbon group.

The pretest was adopted to test the consistency level of stimuli. The participants first had to read product introductions of the carbon–nutrition group and carbon–service group (the order is random). Then they should evaluate the consistency degree of cues. The three items were as follows: “I think the two functions provided by this product are closely related”, “I think the two functions provided by this product are highly similar”, and “I think the two functions provided by this product are the same” (1 = not at all, 7 = very much so) (α = 0.929). According to the pretest (63 participants), there was a significant difference in the consistency degree of two labels (Mcarbon-nutrition = 4.026, SD = 1.777 vs. Mcarbon-service = 3.587, SD = 1.743, t(62) = 2.828, *p* = 0.006). Therefore, service and carbon labels could be used for testing the low consistency level of joint labels, and nutrition and carbon labels could be used for testing the high consistency level of joint labels.

#### 3.4.2. Procedure and Materials

**Figure 9:**
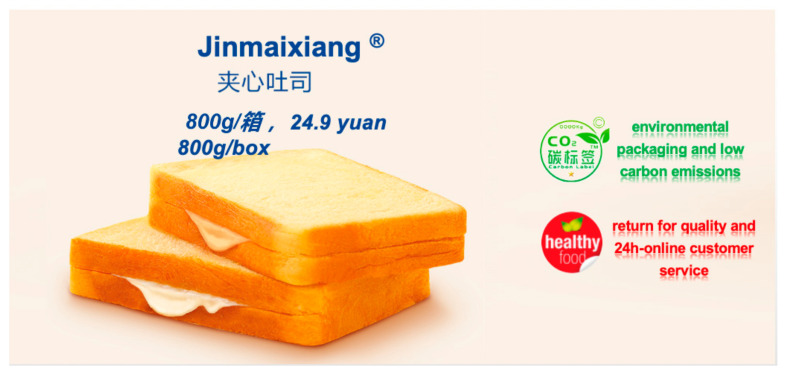

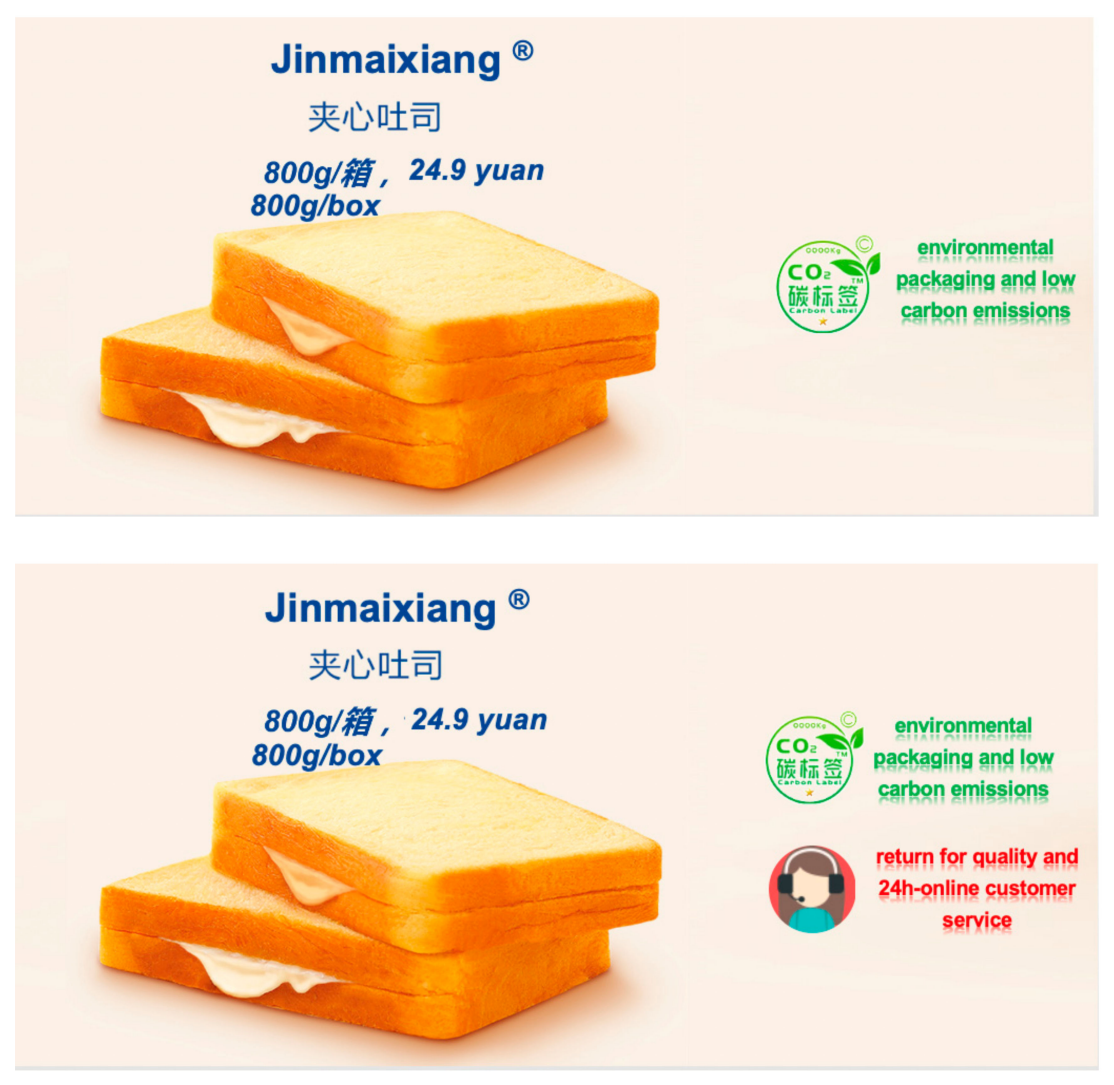
Labels in study 4.

In a formal experiment, participants were randomly assigned to one of these three groups and should read the material of this sandwich toast, and accordingly evaluate and score. The background story of experiment was a fictitious sandwich toast of “Jinmaixiang” (800g/box, 24.9 yuan). For the nutrition label, the attribute was described as “high-quality milk powder and more protein”. For the carbon label, the attribute was described as “environmental packaging and low carbon emissions”. In the service label, the attribute of the products was described as “return for quality and 24h-online customer service.” The nutrition–carbon group added both nutrition and carbon labels, the individual carbon group only added the carbon label, and the service–carbon group added the service and carbon labels (Figure 9). Participants were asked to read the product materials and evaluate and score accordingly.

For the sandwich toast product, they firstly evaluated the resource allocation and anticipated enjoyment for product labels. Their purchase intention and word-of-mouth were also indicated, and finally this study evaluated the conceptual fluency. Items of conceptual fluency include “After reading the above introduction, I can clearly understand the advantages and selling points of the toast”, “I can easily confirm the product introduction of this toast to consumers”, and “I can describe the benefits of this toast to consumers” (1 = not at all, 7 = very much so). These measurement items were adjusted based on Sirianni et al. [68].

#### 3.4.3. Results

Manipulation check. There was significant difference in consistency level of two labels (M_carbon-nutrition_ = 4.811, SD = 1.611 vs. M_carbon-servicet_ = 4.099, SD = 1.738, t(89) = 2.021, *p* = 0.046). This proved that there were significant differences between the two groups in the properties of consistency and that the manipulation of the stimulus was successful. Further, in the individual carbon group, the consistency level of labels was tested by these items, including “If the company needs to add a nutrition label or service label to the toast, which one do you think is more relevant to the low carbon label” (a random order of choice). Finally, 97.78% participants chose the nutrition label. Therefore, in the following article, carbon–nutrition is referred to as joint-con and carbon–servicet as joint-incon. The results provide support for Hypothesis 4.

**Figure 10:**
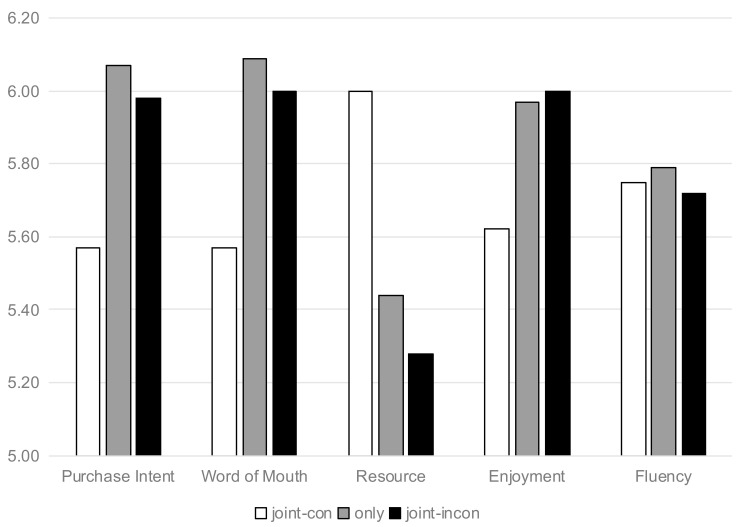
Backfire on the effect of joint positive cues in a different situation of consistency of cues (Sandwich Toast).

Purchase Intention. The results of study 4 were depicted in Figure 10. A one-way ANOVA revealed the backfire on the effect of joint labels (vs. individual) on the purchase intention among three groups (F(2133) = 4.085, *p* = 0.019). Consumers has lower purchase intention when the food labels were highly consistent (M_only_ = 6.07, SD = 0.751 vs. M_joint-con_ = 5.57, SD = 1.108, t(87) = 2.49, *p* = 0.015). However, consumers’ purchase intention had no significant difference when the food labels had low consistency (M_only_ = 6.07, SD = 0.751 vs. M_joint-incon_ = 5.98, SD = 0.737, t(90) = 0.567, *p* > 0.1). In the same way, consumers have lower word-of-mouth (t(87) = 2.493, *p* = 0.015) when the food labels were high in consistency.

Resource Allocation. By a one-way ANOVA, the significant backfire on the effect was revealed between the positive and highly consistent joint labels (F(2133) = 4.645, and *p* = 0.011). Consumers perceived higher resource allocation when the food labels were highly consistent (M_only_ = 5.44, SD = 1.099 vs. M_joint-con_ = 6, SD = 0.778, t(87) = 2.748, *p* = 0.007). However, if joint positive labels had low consistency, the resource allocation had no significant difference (M_only_ = 5.44, SD = 1.099 vs. M_joint-incon_ = 5.28, SD = 1.514, t(90) = 0.606, *p* > 0.1).

Anticipated Enjoyment. A significant backfire on the effect was revealed between the positive and highly consistent joint labels (F(2133) = 3.542, and *p* = 0.032). Consumers had lower anticipated enjoyment when the food labels were highly consistent (M_only_ = 5.97, SD = 0.681 vs. M_joint-con_ = 5.621, SD = 0.858, t(87) = 2.128, *p* = 0.036). However, if joint positive labels had low consistency, consumers’ anticipated enjoyment had no significant difference (M_only_ = 5.97, SD = 0.681 vs. M_joint-incon_ = 6, SD = 0.699, t(90) = 0.206, *p* > 0.1).

Mediation Analysis. A serial mediation analysis was further conducted, including resource allocation and anticipated enjoyment. When the dependent variable was purchase intention, the comparison of highly consistent group was made. The full serial mediation model was significant (indirect effect = −0.0881, SE = 0.0542, 95% CI = −0.2169 to −0.0109), and the “reverse” model was not significant (indirect effect = 0.0207, SE = 0.0179, 95% CI = −0.0024 to 0.0661). However, from the comparison of low-consistency group, this model was not significant (indirect effect = −0.0032, SE = 0.0102, 95% CI = −0.0298 to 0.0148). Similarly, the dependent variable was replaced with word-of-mouth. The full serial mediation model was significant (indirect effect = −0.0925, SE = 0.0505, 95% CI = −0.2052 to −0.009).

Conceptual Fluency. Three items related to the conceptual fluency were highly correlated (α = 0.802). The conceptual fluency was not significant under the conditions of three scenarios (F(2133) = 0.1, *p* > 0.5, M_joint-con_ = 5.75, SD = 0.87, versvs.us M_only_ = 5.79, SD = 0.77 and M_joint-incon_ = 5.72, SD = 0.82). In addition, from a bootstrap analysis with 5000 samples using Model 4, it was indicated that the mediation model of conceptual fluency was not significant either (indirect effect = −0.0079, SE = 0.0416, 95% CI = −0.0948 to 0.0694).

Control Variable. The background knowledge of COO or organic label has no significant interaction between the labels and the zero-sum bias (F(1125) = 0.198, *p* > 0.1). Four conditions were coded, i.e., 11 = joint labels and high zero-sum bias, 00 = individual organic label and low zero-sum bias, 10 = joint labels and low zero-sum bias, and 01 = individual organic label and high zero-sum bias. The mean values between groups were as follows: M_11_ = 5.56, SD = 0.963, M_00_ = 5.35, SD = 1.07, M_10_ = 5.3, SD = 1.171, and M_01_ = 5.5, SD = 1.308.

## 4. General Discussion

We conducted four experiments to answer the question, and results showed that nutrition label and low-carbon label work simultaneous may attenuate the consumer’s preference for food product compared with single label (Hypothesis 1). We refer to the effect as joint backfire, which is driven by two mediators: Zero-sum bias [50] and enjoyment motivation [47]. Facing nutrition and carbon labels simultaneously, people will infer that resources were allocated to healthy and environmental aspects so they have a lower evaluation on taste of the food (Hypothesis 2). The perceived discount in taste led to a negative effect on preference of consumers. The joint backfire effect was exerted in people with a higher level of zero-sum bias (Hypothesis 3). Specifically, we examined consistency of cues [57], and results demonstrated that consumers’ purchase intentions have no significant difference when labels are inconsistent, which suggested that consistency is the boundary of the joint backfire on the effect (Hypothesis 4). At the same time, by testing these four hypotheses, we have three further research conclusions to discuss.

First of all, we contribute to determining the joint effect of the nutrition label and the carbon label, which is one of crucial strategies to tackle unhealthy diets and environment problems [28,69,70,71] and is also the future direction of food attribute research. On the one hand, most studies only presented the isolated effects of labels [21], and limited literature has examined the joint effect of multi attributes of food. This is not conducive to the in-depth study of food properties. Therefore, the study on the joint effect of attributes in this paper provides a new research vision for future food research. In particular, two different positive attributes can be studied together to explore the interaction between them, so as to choose the most appropriate combination to improve the preference of food sustainable attributes to consumers. On the other hand, although scholars analyzed the joint effect of different labels (or attributes), they have come to conflicting conclusions that the combined effect has an alternative or complementary effect on food purchase [26,27,43]. Many previous studies suggested that multi attributes can enhance incremental utility [34,35]. However, recent studies have also shown that rich information can lead to negative purchase decisions when the information goes over the threshold [15]. Our results support the second conclusion, which is that the combined effect has an alternative effect on food purchase. To be specific, labels for two dimensions of sustainability may discount the positive effect on consumers’ preference compared with a single label. This further supplements the previous research on the joint effect of positive labels [72]. All in all, this is the latest research achievement of an in-depth analysis of the mechanism behind the negative effect of joint labeling, which can provide strong evidence and reference for solving the contradictory conclusions between different scholars and the joint labeling effect research in the future.

Secondly, we have conducted in-depth analysis on the psychological mechanism behind the adverse effect of joint labeling and obtained a serial mediator model. The psychological mechanisms behind food labeling are rarely involved in research on food labeling [73]. Meanwhile, how the joint positive labels affect consumers’ cognition has been scarcely studied [39]. In particular, the studies that show the negative effects of joint labeling do not provide the underlying mechanism behind it [40]. Therefore, this study, after absorbing the related theories of psychology and consumer behavior, combining zero-sum theory [39] and anticipated enjoyment [47], provides an in-depth insight into this process. The process is as follows: Based on zero-sum bias, it is inferred by consumers that the food has mores resource allocated to positive attributes than to taste, and further, resources spent on the positive attributes indicate a lower anticipated enjoyment from food. In the general context of pursuing food taste, consumers’ low anticipated enjoyment will lead to a decline in purchasing intention. This is the first time that a study has teased out the consumer psychological mechanism of the adverse effect of joint labeling and to prove it experimentally.

Finally, we determine the boundary condition of the joint backfire. The discovery of boundary conditions for the joint effect is beneficial to improve the level of label management in the field of practice and to explain the contradictory conclusions of different scholars on the joint effect. There is a high possibility that the conclusion of positive effect of joint labels [36,37] is produced because they are at the other end of the boundary conditions in the experiment or design, such as the low zero-sum bias of the subjects in the experiment, or the inconsistency in dimensions of the two labels given by the experimental design, etc. At the same time, the zero-sum bias is adopted to further analyze the difference in zero-sum bias among consumers [50]. This important consumer trait was not mentioned in a series of previous study [15,38], and here we demonstrate that zero-sum bias is a consumer trait that can easily affect the final results of the study. In addition, consistency level of labels is used to find the boundary for the joint inverse effect of positive labels, and to also coordinate and explain the contradiction existing in previous studies [27,43]. To be specific, the consistency level of labels enhances the zero-sum bias on resource allocating conception and infer poorer taste of food. Thus, these two moderating variables greatly supplemented the theoretical boundary of main effect.

### 4.1. Theoretical Contribution and Practical Implication

The conclusions from this study also have two theoretical implications. The first important theoretical contribution is that the results of this study have extensively demonstrated the effect of joint positive labels on the consumer’s perception in different contexts. The verification of this main effect and its mechanism could promote the development of compensatory inferences in the food field. In previous studies, some scholars have verified the existence of compensatory inferences in other contexts [51], especially the early discovery of “the unhealthy = tasty intuition” [74], which has promoted much research on the food taste and positive attributes. However, the experimental group of the individual label in this study did not indicate that nutrition or carbon labels directly form a situation of bland food. It is proposed that as more and more food producers pay more attention to positive attributes, such as health and low-carbon attributes, consumers will regard moderately positive attributes as a normal attribute of product. It will not trigger the compensation effect under the zero-sum game, but similar effects such as “the unhealthy = tasty intuition” will occur under the condition of over-emphasis on the positive attributes, that is, under the scenario of joint-consistency positive labels. Therefore, this research has a certain promotion influence on the consumer research related on compensatory inferences.

The second theoretical contribution is to enrich the interactions of positive labels in food from the perspective of food labeling research, and to explore the relationship between positive labels and consumers’ internal needs. The consistency level of cues is used to explain why scholars have contradictory conclusions about the joint effect of positive labels. At the same time, it further supported the conclusion “adding consistent quality labels have a decreasing marginal effect” explored by some scholars [72]. In a sense, this research provides a theoretical basis for follow-up research. Future research can further analyze the joint effect between different labels and explore joint forms to eliminate the negative effect. In addition, with the actual requirements of food industry, the single main effect analysis cannot satisfy a deeper understanding of consumers. Therefore, this research provided in-depth insights on the potential psychological mechanism of consumer’s backfire on the effect of joint positive cues and formed a serial mediation mode. At the same time, this psychological mechanism also constituted a dialectical relationship between positive labels and anticipated enjoyment through the resource allocation.

These conclusions, which are contrary to actual practice. have important management implications for food companies, health associations, and environmental protection organizations. Firstly, it is suggested that when creating product selling points, companies should not grant food products too many labels in similar dimensions, as they may lead to lowering consumer’s purchase interest. Therefore, the creation of selling points should take the form of “orthogonal” complementarity. If a company wants to reflect as many differences as possible between its own food and other competitors, it is better to provide labels with different dimensions (large differences in functions), such as low-carbon and after-sales service cues. Secondly, a serial mediation model provides another perspective for enterprises to gain an insight into the inner logical mechanism of consumers. When food companies provide additional positive goals for food, they should consider the pure taste needs of consumers. Positive goals certainly support consumers’ “super-ego”, but consumers are often driven by the original “ego” to make food choices. This contradiction is the “war” between positive labels and anticipated enjoyment, which comes from consumers’ potential zero-sum game thinking. How to grasp the balance between goals and needs of consumers should be paid more attention by food companies. Thirdly, the results of this study also provide a way to manipulate consumers’ zero-sum heuristic, and thus to solve the backfire effect of joint positive labels. In the research, the method of measuring the zero-sum bias is used to control, but in real life, there are many ways to reduce the zero-sum bias of consumers, such as manipulating the regulatory focus through scenarios and stimulating the consumers’ promotion focus thinking. Through the promotion focus, consumers tend to pay more attention to the benefits of products [75].

### 4.2. Future Research Directions

Limitations in this study and the future research directions are as follows: The first problem is that the starting point and experimental scenarios discussed in this study mainly focus on the scenario of consumers buying food separately. That is to say, in terms of purchasing motivation, consumers consider personal needs more. In this case, the possibility of the pursuit for food taste will be greater. If a consumer is a family food decision maker, or buys food for others, will the consumer reduce the decision-making weight of taste in food choice, leading to the disappearance of the main effect of this study? Although this study can expect that taste is still an important influencing factor when choosing food for family or others, more and more studies also have reflected that differences in the identity of buyers will affect buyers’ concerns in the food selection process. For example, parents pay more attention to the nutrition and health when choosing their children’s food [76]. Therefore, in future research, more purchase scenarios should be considered. The research thinking of this study combined with scenarios of family food decision-making and food gifting can enhance the universality of these findings.

The second problem is that although a variety of dependent variable measurements were used for respondents by the situational experiment method, including the purchase intention, perceived value, word-of-mouth publicity, etc., there would be some discrepancies with actual purchase behaviors. In the future, field experiments and selective experiments can be used to enhance the rationality of dependent variable measurement, and to strengthen the external validity of research conclusions. There may be more factors considered in the actual purchase process.

## 5. Conclusions

Concluding, this study investigates the effect of nutrition label and carbon label within individual and joint situations, through a serial mediation of zero-sum bias and anticipated enjoyment in sustainable consumption. This research then detects the boundary condition to the joint effect of food labels. Finally, this study suggests that similar labels should be avoided in the same front-of-pack of food, or manufacturers need to use ads to bring down consumers ‘zero-sum bias and reduce the joint backfire on the effect.

## Figures and Tables

**Table 1:** Socio-demographics of consumers in Study 1–4.

Socio-Demographic Indicators		Study 1	Study 2	Study 3	Study 4
Variable	Definitions	Percentage	Percentage	Percentage	Percentage
Gender	Male	47.1%	42.2%	41.1%	48.5%
	Female	52.9%	57.8%	58.9%	51.5%
Age	≤20 years old	5.1%	6.1%	4.7%	10.3%
	21–30 years old	76.5%	72.1%	76.7%	66.9%
	31–40 years old	17.7%	20.4%	17.8%	18.4%
	≥41 years old	0.7%	1.4%	0.9%	4.4%
Education	high school degree	0.7%	2.0%	2.3%	2.2%
	junior college	13.2%	18.4%	20.2%	16.9%
	bachelor’s degree	82.4%	72.1%	74.4%	75.7%
	post-graduate degree	3.7%	7.5%	3.1%	5.1%
Valid sample size		136	147	129	136
